# Co-feeding Transmission and Its Contribution to the Perpetuation of the Lyme Disease Spirochete *Borrelia afzelii* (In Reply)

**DOI:** 10.3201/eid0907.030116

**Published:** 2003-07

**Authors:** Sarah Randolph, Lisa Gern

**Affiliations:** * University of Oxford, Oxford, United Kingdom; † Université de Neuchâtel, Neuchâtel, Switzerland

Richter et al. (*1*) have asked an important question: To what extent does the transmission of nonsystemic infections of the Lyme borreliosis spirochete (*Borrelia afzelii)* between co-feeding nymphal and larval *Ixodes ricinus* ticks apply to natural tick infestations on wild rodents? The authors conclude that the transmission of infections 3 days after inoculation by tick bite is >100 times less efficient than the transmission of infections that have lasted at least 14 days. That answer depends on a critical calculation based on experimental results combined with field observations. Unfortunately, this calculation is incorrect by a factor of approximately 20.

When hairless laboratory mice were restrained within wire mesh tubes and larvae were allowed to attach at random over their bodies, 13.6% of these larvae became infected with *B. afzelii* if they fed 3 days after the attachment of a single infected nymph (i.e., transmission probability of 0.136, as used below). By contrast, 85.4% of larvae that fed 14 days after the nymph became infected (*1*). At three sites in Germany and France, over the period April–October in each of the years from 1993 through 1995, 17.6% of mice (*Apodemus flavicollis* and *A. sylvaticus*) and voles (*Clethrionomys glareolus*) fed larval and nymphal ticks together, while 1.5% fed nymphs alone. Of these nymphs, 26.4% were infected with *B. burgdorferi* s.l. before attachment. The probability of a larva’s acquiring an infection equals the product of 1) the probability of transmission from host to larva and 2) the probability of the host’s being infected while the larva feeds via an infected nymphal tick bite. For a short-lived (3-day) infection, the probability is 0.136 × 0.176 × 0.264 = 0.0063; for longer-lived (14-day) infections, the probability is 0.854 × (0.176 + 0.015) × 0.264 = 0.0431. The ratio is therefore 1:6.8. Richter et al. erroneously concluded that the ratio was 1:116 because they did not take into account the probability of wild rodents’ acquiring a long-lived, ”systemic” infection; the authors assumed the probability was 1. A greater proportion of garden dormice (*Eliomys quercinus*) carried ticks and so would yield much higher transmission probabilities but in almost the same ratio, 1:6.4.

In fact, how much of the increase from 13.6% transmission at day 3 to 85.4% at day 14 was due to the development of systemic infections (i.e., disseminated to parts of the hosts’ bodies >2 cm from the infected tick bite) is not clear because the feeding sites of the larvae attached ad libitum on the hairless mice were not reported. In the original discovery of co-feeding transmission of *B. burgdorferi* s.l (*2*), the infection prevalence in larvae feeding close to infected nymphs increased from 33% on day 2 to 96% on day 11 and 100% on day 14 (*3*) in the demonstrated absence of a systemic infection. Mice skin and ticks feeding at distant sites remained uninfected. Only after day 14 had a systemic infection developed (*2*). Because spirochetes are not transmitted to the host until at least 17.6 h after an infected nymph starts feeding (*4*–*6*) and then disseminate only slowly from the feeding site (*7*), co-feeding in space rather than in time is the crucial feature in Lyme borreliosis (*2*,*8*) (so-called “extended co-feeding”[*3*]). Larvae that attach to hosts simultaneously with infected nymphs rarely acquire spirochetes (*1*,*9*), wherever they attach. This pattern is distinct from the more immediate and short-lived co-feeding transmission of tickborne viruses (*10*–*12*). In both cases, however, the key feature is a nonsystemic infection.

Despite the uncertainties in Richter et al.’s study, their corrected ratio is very similar to that (1:5.7) calculated (*3*) with a “synthetic model . . . based on major assumed parameters” (sic) (*1*). In that model we assumed that 50% of larvae were likely to be feeding within 1 to 2 cm of any infected nymph, the distance over which co-feeding ticks can pick up nonsystemic infections (*1*,*2*) because in the wild very few rodents carry nymphs in the absence of larvae (*1*,*13*), and >95% of all immature stage ticks feed in aggregations, mostly on the ears and also around the eyes or on the snouts of mice and voles. Considerable risks exist in using laboratory experimental results to quantify the epidemiologic importance of nonsystemic infections in the wild because of differences between host species, unnatural spatial distributions of introduced ticks on hosts, and the subtleties of natural tick-host relationships. Coincident aggregated distributions of larvae and nymphs among their rodent hosts, whereby the same individual hosts carry the largest numbers of both stages, increase the number of larvae co-feeding with any infected nymph, and so augment the potential amplification of infection prevalence in ticks (*13*). Nevertheless, in the case of rodents, nonsystemic infections are soon rendered redundant by the much longer lived systemic infections. In contrast, in the case of host species in which systemic infections do not develop, the transmission of nonsystemic infections between co-feeding ticks is the only way in which infection prevalence can be amplified in feeding ticks. Field data suggest that this route of transmission occurs on wild Sika deer (*Cervus nippon*) (*14*). Natural experimental systems have confirmed that on sheep this transmission pathway exists and is sufficient alone to maintain enzootic cycles of Lyme borreliosis (*8*).
